# Barriers to Effective Doctor-Patient Relationship Based on PRECEDE PROCEED Model

**DOI:** 10.5539/gjhs.v7n6p24

**Published:** 2015-03-25

**Authors:** Saeideh Ghaffarifar, Fazlollah Ghofranipour, Fazlollah Ahmadi, Manouchehr Khoshbaten

**Affiliations:** 1 Department of Health Education and Health Promotion, Faculty of Medical Sciences, Tarbiat Modares University, Tehran, Iran; 2 Department of Nursing, Faculty of Medical Sciences, Tarbiat Modares University, Tehran, Iran; 3 Medical Education Research Center, Faculty of Medicine, Tabriz University of Medical Sciences, Tabriz, Iran

**Keywords:** doctor-patient relationship, barriers, qualitative study, PRECEDE PROCEED

## Abstract

**Objective:**

This study intends to investigate interns and faculty members’ insights into constructing relationship between physicians and patients at 3 more accredited Iranian universities of medical sciences.

**Method:**

Applying PRECEDE PROCEED model, semi-structured interviews were completed with 7 interns and 14 faculty members and two themes were emerged from directed content analysis. The meaning units of the first theme, barriers to effective doctor-patient relationship, are discussed in this paper.

**Results:**

According to the participants, building doctor-patient relationship is influenced by many contextual and regulatory factors as well as content, process and perceptual skills of physicians.

**Conclusions:**

Faculty and curriculum development, as well as foundation of the department of communication skills at medical schools are recommended to eliminate the impact of poor communication on patients’ satisfaction and physicians’ self-efficacy specific to their communication skills.

**Practice Implications:**

Applying theories and models of health education and health promotion, researchers and educators can use the most predictive constructs of theories to design and implement effective interventions.

## 1. Introduction

Building effective doctor-patient relationship as the crucial part of a successful medical care (Shumway & Harden, 2003) is one of the most complicated professional responsibilities of physicians because they should simultaneously gather and process patient information in order to share patients in decision making and increase their satisfaction and compliance with medical plans (S. M. Kurtz, 2002; Teutsch, 2003).

Despite worldwide emphasizing on such a great social accountability of physicians, teaching doctor - patient relationship has not been integrated formally into the curriculum of many medical schools (Beran, 2012; Butalid, Bensing & Verhaak, 2014). Even in some medical universities, the very invaluable efforts to reform medical curriculums design and implement communication skills programs have failed to yield desired outcomes and physicians do not appear to build effective doctor-patient relationships professionally (Ghaffarifar, Khoshbaten, Ghofranipour & Kompani, 2013).

Furthermore, internationally, patient complaints about doctors’ communication skills are recorded at the top of the content analyzed complaint lists (Laidlaw, Kaufman, Macleod, Sargeant & Langille, 2001; Montini, Noble & Stelfox, 2008).

Aiming to analyze the situation, some researchers have investigated medical students and faculty members’ perceptions of learning and teaching communication skills. Yet they have not applied theories and models of Health Education and Health Promotion (HEHP) to explore students and faculty members’ perceptions and experiences towards doctor-patient relationship.

**Figure 1:**
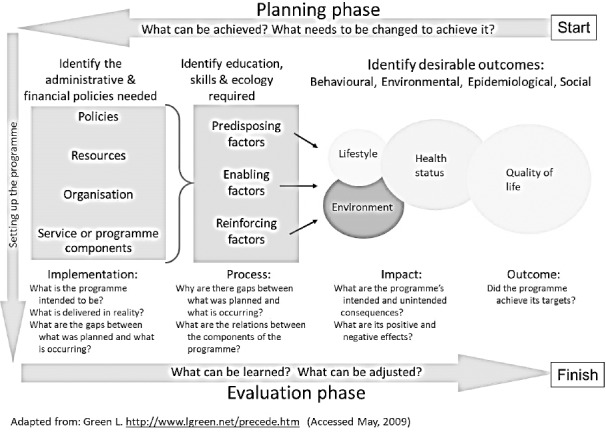
Different phases of PRECED PROCEED model

Applying theories and models of HEHP lets health researchers and educators to have a deep and comprehensive understanding of a health behavior; for example, building effective doctor-patient relationship. PRECEDE PROCEED is one of the most popular planning models in health education. This model provides researchers a conceptual framework, based on the principle of participation of the target group in planning and designing the intervention and evaluating of its impacts and outcomes. Hence, doing a situational analysis in the first phase of it, as well as its flexibility and its ability to embody the pertinent constructs of the other theories and models of health education and health promotion everywhere and every time during the research, represents it as the most table model to evaluate interns’ communication skills and implement the most efficient and effective intervention in order to improve interns’ doctor-patient relationships. The phases (steps) of PRECEDE PROCEED model are introduced in Figure 1.

Hence, finding out the most predictive construct(s) of theories and models of HEHP, they can design the most specific and unique educational intervention(s) in order to address the existing gaps (Glanz, Rimer, & Viswanath, 2008; Sharma & Romas, 2011).

With regard to the social accountability of medical schools for management of doctor-patient relationship problems and reduction in patient complaints and also with regard to the utmost importance of applying theories and models of HEHP in educational asset mapping and needs assessment, this qualitative study as a part of a larger study, applying PRECEDE PROCEED model(Green & Kreuter, 2005), explores medical interns and faculty members’ perspectives and experiences about doctor-patient relationship and discusses the barriers to effective doctor-patient relationship at 3 more accredited Iranian universities of medical sciences, Tehran, Shahid Beheshti and Tabriz.

## 2. Method

### 2.1 Context of Training

The general medical curriculum at Iranian universities of medical sciences is spread over 7 years and all interns are being given practical training under teaching faculty members’ supervision during the last 18 months of their studying at medical schools. Communication skills programs are hospital-based and building doctor-patient relationship is considered as a non-core competency in most medical schools; however, instructional strategies vary among different schools. With a diverse range between one-day workshop and three-credit formal course, different programs are evaluated with a wide variety of assessment methods.

### 2.2 Participants

Participants of this research were interns and teaching faculty members of Iranian universities of medical sciences, Tehran, Shahid Beheshti and Tabriz. Willingness to be interviewed was the main inclusion criteria for all participants. Furthermore, applying PRECEDE PROCEED model in the research, a sampling based on a maximum variant approach in terms of academic position of faculty members and the level of their involvement in teaching doctor-patient relationship programs was a must.

### 2.3 Data Collection

The ethical approval for this research was obtained from Tarbiat Modares University’s Ethics Committee in 2013. A purposive sample of teaching faculty members and a convenience sample of interns were recruited to be interviewed. The first faculty member, who was a vice chancellor for education, introduced the second key informant based on his/her role in teaching communication skills program and recruiting the rest of the faculty members was continued by the snowball method. In order to elicit data and explore faculty members and interns’ perceptions and experiences about doctor-patient relationship, SGH conducted semi-structured, 40 to 60 minute deep interviews. Open-ended questions were designed based on the objectives of phase 1 in PRECEDE PROCEED model. Participants were already informed of both the objectives of the study and confidentiality of their information over the phone. Being informed about their right to withdraw from participation at any time, participants let SG know about their availability for interview at any convenient time and place for them. They openly shared their perceptions and experiences in reply to the structured and open-ended questions of the research, which were followed by targeted and probing questions upon the case. Identifying the themes and achieving information saturation, interviews were discontinued.

### 2.4 Data Analysis

Directed content analysis method was concurrently done with data collection. In this research, the framework of PRECED PROCEED model was conceptually used to identify initial coding categories. Hence, using the predetermined coding categories, highlighted passages were coded and themes were emerged from the fully transcribed interviews. Frequency of meaning codes was calculated in order to compare the rank order of the codes and report the incidence of the codes which represent each theme.

In addition to source triangulation based on maximum variance sampling, in order to establish credibility of findings from interviews participants were asked to confirm the preliminary meaning units (member check) and in order to increase the accuracy of predetermined categories and achieve unbiased results, an auditor review was used (peer check).

## 3. Results

Information saturation was achieved by participation of 14 faculty members and 7 interns. The majority of participants were male (n = 15, 71.4%). Table 1 shows demographics of the interviewees who expressed their perceptions and experiences about building effective doctor-patient relationship at 3 Iranian universities of medical sciences.

**Table 1:** Demographics of interviewees who expressed their perceptions and experiences about building effective doctor-patient relationship at 3 Iranian universities of medical sciences*

Demographic	Number
Interviews by University	
University A	6
University B	8
University C	7
Gender of participants	
Female	6
Male	15
Academic position*	
Instructor of the communication skills program	4
Vice Chancellor for education	3
Head of Education Development Center	2
Head of Education Development Office	3
Head of Medical Education Department	2
Person in charge for Education Committee	1
Merely Faculty member	5
Intern	7
Specialties represented	
Internal Medicine	3
Psychiatry	1
Infectious Disease	1
Urology	1
Pathology	1
Neurology	1
Medical education	3
General practice	3

* some faculty members had more than one academic position.

Two themes related to learning and teaching doctor-patient relationship were identified from the directed content analysis of transcribed interviews. This paper discusses only the condensed meaning units of the first theme, barriers to building effective doctor- patient relationship. Barriers to effective doctor-patient relationship were listed as 49 and 15 condensed meaning units based on faculty members and interns’ points of view respectively. Table 2 and Table 3 demonstrate 10 most common condensed meaning units from faculty members and interns’ interviews based on the incidence of the codes which represent the theme of barriers to effective doctor-patient relationship.

**Table 2:** Incidence of condensed meaning units of barriers to effective doctor-patient relationship from faculty members’ interviews

Condensed meaning unit	Number	Percent
No supervision of patient education	13	92.9
Overcrowded wards	12	85.7
Instructors’ poor communication skills	12	85.7
No payment for education	12	85.7
Paternalistic attitude of physicians towards patients	11	73.3
Not repeating of communication skills program at different stages of studying	11	73.3
Focus on content rather than the process of instruction	10	71.4
Using medical jargon	9	64.3
Inappropriate context of education	9	64.3
Not evaluating students’ communication skills in summative assessments	7	50.0

10 most common barriers from all 49 barriers, which were emerged from 14 faculty members’ interviews.

**Table 3:** Incidence of condensed meaning units of barriers to effective doctor-patient relationship from interns’ interviews

Condensed meaning unit	Number	Percent
Overcrowded wards	7	100
Not evaluating students’ communication skills in summative assessments	7	100
Inappropriate context of education	6	85.7
Bing on duty and weary at many nights per month	6	85.7
Not repeating of communication skills program at different stages of studying	5	71.7
Instructors’ poor communication skills	5	71.7
Focus on content rather than the process of instruction	5	71.7
No tendency of patients to be visited by interns	5	71.7
Distress and anxiety to patient education	5	71.7
Having some non-educational conflicting roles	5	71.7

10 most common barriers from all 15 barriers, which were emerged from 7 interns’ interviews

In all, a general consensus among participants was clearly elicited that physicians’ content, process and perceptual skills are the main barriers of establishing effective doctor-patient relationship. Table 4 reveals verbatim quotations, which support the theme of barriers to effective doctor-patient relationship.

**Table 4:** Verbatim quotations, which support the theme of barriers to effective doctor-patient relationship

1. Many patients are hospitalized in every shift, so physicians can only take histories, not more. You need to divide your time among many needful patients. Therefore, it is not possible to educate patients and share them in decision making in such busy situations (Interns, faculty members).
2. I have visited many patients, who were uncomfortable to tell me about their complaints. They were always looking around to check that if other patients and their attendants hear them or not! Well, how can I build a good relationship with patients without having their privacy in mind? (Interns, faculty members).
3. Unfortunately, authorities do not supervise physicians’ communication skills… physicians are very smart and do not allocate their time to the subjects and activities that are never evaluated because there is not any penalty or even incentives regarding communication skills! (Interns, faculty members).
4. Most university professors are really bad at communicating with patients…They themselves need training before supervising students’ skills and providing feedback to them… (Interns, faculty members).
5. Unfortunately, some of faculty members with poor communication skills, who are the best in their specialties, become students’ role models!! Their students prefer to develop their technical knowledge and skills the same as their role models, in order to be famous like them…so, other professors’ endeavors to increase students’ motivation to learn about communication skills fail to succeed! (Interns, faculty members).
6. There is no opportunity to work in teams because every resident or intern has follow to his/her service commitment which never finishes…usually, interns do not receive feedback on their communication skills from their classmates or residents. If medical students had the opportunity to share their experiences in small groups, they would be able to learn from each other’s mistakes (interns, faculty members).
7. In workshops and classes, medical students learn what they should do…for example: a physician should greet patient…should introduce her/himself…should present information in a clear way…but they do not learn about how they can do these tasks, they seldom have an experiential course to practice them (interns, faculty members).
8. Most visits are doctor-centered and physicians usually think that patients’ comments are not useful, so they do not let patients to tell everything…they always interrupt patients…even, some patients think that their physician is like their God and knows everything and they do not need to say everything(interns, faculty members).
9. Medical students attend communication skills programs only one time around their clerkship…even, if they want to practice those skills, they usually do not remember what they have already learnt…(interns, faculty members).
10. Talking with patients and their examination is stressful, when your professor observes you. It seems, you have forgotten anything at that time …I am always regretful after my visits in the presence of my teachers. I think, if I had controlled my feelings, I would have presented my skills better and received a good feedback(interns)

## 4. Discussion

In this research, more than 85 percent of participants declared that visiting patients in overcrowded wards was a pivotal barrier of building effective doctor-patient relationship. Some interns and faculty members blamed hospitalization of excessively high number of patients per night for not allocating enough time to establish efficient relationship with patients (Table 4, quote 1). Other interns and faculty members criticized visiting several patients in a common room for intruding into patients’ private situations (Table 4, quote 2). Similarly, in Rees and associates’ study, medical students declared that their instructors did not let them to build effective relationships because of time limitations (Rees, Sheard, & McPherson, 2004). Ghaffarifar and colleagues’ study, in which the average visit time of out- patients at an Iranian academic medical center was 7 to 13 minutes, (Ghaffarifar, Ghojazadeh, Alizadeh, Ghaffari & Sadeghi-Ghyassi, 2012) verifies the influential significance of spending enough time to build effective relationships with patients. Hence, cutting the remuneration of the physicians for the visits beyond the ratified number of patients per hour or day is suggested as a practical and efficient way to tackle the problems emerging from time limitations in overcrowded academic medical centers.

According to 93 percent of faculty members, people in charge of education in medical schools did not supervise the process of patient education (Table 4, quote 3) because they themselves had poor communication skills (Table 4, quote 4). Moreover, 71 percent of them concluded that poor communication skills of instructors impressively influence medical students’ motivation to establish effective relationships with patients (Table 4, quote 5) and according to 64 percent of faculty members, the context of education was not appropriate enough to set up successful relationships(Table 4, quote 6). In order to manage these crucial barriers, educational planners should give preference to investment in continuing faculty development (Lang, Everett, McGowen & Bennard, 2000; Loureiro, Severo, Bettencourt & Ferreira, 2011) and strengthening of educational infrastructure. Medical curriculums should be reformed (Hauer, Carney, Chang & Satterfield, 2012) and students should be involved in active learning methods. These recommendations are in consistence with Berkhof and colleagues’ conclusions that compared to passive learning methods, being involved in active learning methods, like discussions in small groups, receiving constructive feedback and observing good role models, results in better outcomes in terms of triumphantly communicating with patients(Berkhof, van Rijssen, Schellart, Anema & van der Beek, 2011).

Mainly focusing on the content of communication skills programs rather than concerning the process of teaching and learning of the content by both instructional designers and educators was a substantial communication barrier based on perspectives of 71 percent of participants (Table 4, quote 7). As Kurtz and colleagues recommend, “marrying content and process” (S. Kurtz, Silverman, Benson & Draper, 2003) should be regarded as one of the most important educational objectives in formulating the course plan of communication skills and faculty members should develop their own competencies in teaching the combined content and process of building effective relationships.

Paternalistic attitude of most physicians, which in turn, resulted in passive roles of patients, was another obstacle to establishing productive doctor-patient relationship, according to 73 percent of participants (Table 4, quote 8). Bertakis and associates’ study verifies this finding. In their study, patients were more satisfied with the visits, in which they had active roles and were able to openly ask their questions and share their opinions(Bertakis, Roter, & Putnam, 1991). In order to successfully deal with this cultural barrier to doctor- patient relationship, medical schools should provide faculty members and students with opportunities, in which they can practice shared decision making and empathetic behavior towards their patients and in order to criticize being paternalistic and gradually modify the existent dominant role of physicians, discussing some videotaped patient visits and providing feedback on them by peers and instructors are recommended.

Communication skills programs were presented at only one stage of medical studying based on declarations of 73.3 percent of participants. They believed that not repeating of the programs at different phases of their studying at medical schools decreases their chance to receive feedback on their performance in communicating with patients, which in turn, reduces internalization of both the content and practical applications of the programs (Table 4, quote 9). For this reason, in order to achieve better outcomes and maintain behavioral capabilities of medical students in terms of their communication skills, repeating communication skills programs at rational intervals combined with providing continual constructive feedback on students’ communication skills is proposed. Aspegren’s review on 24 systematic reviews about “teaching and learning communication skills in medicine” (Aspegren, 1999) and Merckaert and colleagues’ study about communication skills training (Merckaert, Libert, & Razavi, 2005) verify this suggestion. Likewise, the finding of Gysel and colleagues’ study, which emphasis on integrating theoretical and practical aspects of communication skills programs and planning long and learner-directed programs (Gysels, Richardson, & Higginson, 2004) is also in agreement with this recommendation.

Organizing the department of communication skills at medical schools is recommended because the stewardship of the communication skills programs by specialized knowledge of an independent department allows medical schools to employ efficient solutions to most of the barriers to doctor-patient relationship.

For 71.7 percent of interns of this study, building relationship with patients in the presence of their instructors and teaching faculty members was stressful (Table 4, quote 10). According to the theory of self-efficacy, feeling stress negatively impacts peoples’ confidence in their capability to proceed with a behavior (Bandura, 2006). So, in order to lessen harmful effects of feeling anxiety on medical students’ self-efficacy specific to communication skills and reduce its subsequent unpleasant influence on the efficacy of communication skills programs (Ammentorp, Sabroe, Kofoed & Mainz, 2007), plumbing underlying causes of experiencing such stresses is suggested. In this regard, Gysels and colleagues believe that educational programs should educate students about adopting positive attitude and beliefs in efficient management of emotional situations (Gysels, et al., 2004).

## 5. Conclusions

This study was conducted to appreciate problems of building a good rapport between doctors and patients, using the perceptions of interns and faculty members. Content analysis indicated that many contextual, individual, educational and regulatory factors affect creating effective doctor-patient relationship and physicians’ communication skills can be improved by curriculum and faculty development and establishment of the department of communication skills at medical schools. Applying PRECEDE PROCEED as a planning model, allowed researchers to attract participants’ more cooperation in finding the key solutions to the problem of physicians’ poor communication skills. In other words, content analyzing of participants’ information revealed a theory which better described influential factors on doctor-patient relationship. Furthermore, finding most predictive construct(s) of that emerged theory allowed researchers to design and implement an appropriate educational intervention easily, at the next phase of PRECEDE PROCEED (the results of these applications have been discussed in another manuscript).

## References

[ref1] Ammentorp J, Sabroe S, Kofoed P.-E, Mainz J (2007). The effect of training in communication skills on medical doctors’ and nurses’ self-efficacy: A randomized controlled trial. Patient Education and Counseling.

[ref2] Aspegren K (1999). BEME Guide. No. 2: Teaching and learning communication skills in medicine-a review with quality grading of articles. Medical teacher.

[ref3] Bandura A (2006). Guide for constructing self-efficacy scales. Self-efficacy beliefs of adolescents.

[ref4] Beran T (2012). Advances in Medical Education: The Importance of Communication and Collaboration. Canadian Medical Education Journal.

[ref5] Berkhof M, van Rijssen H. J, Schellart A. J, Anema J. R, van der Beek A. J (2011). Effective training strategies for teaching communication skills to physicians: An overview of systematic reviews. Patient Education and Counseling.

[ref6] Bertakis K. D, Roter D, Putnam S. M (1991). The relationship of physician medical interview style to patient satisfaction. The Journal of Family Practice.

[ref7] Butalid L, Bensing J. M, Verhaak P. F (2014). Talking about psychosocial problems: An observational study on changes in doctor–patient communication in general practice between 1977 and 2008. Patient Education and Counseling.

[ref8] Ghaffarifar S, Ghojazadeh M, Alizadeh M, Ghaffari M. R, Sadeghi-Ghyassi F (2012). An Academic Medical Center: A Customized Strategy to Overcome the Shortcomings of Interns’ Ambulatory Education. Shiraz E-Medical Journal.

[ref9] Ghaffarifar S, Khoshbaten M, Ghofranipour F, Kompani J (2013). Residents’ Communication Skills Evaluation by OSCE: What Changes Should be done in Educational System?. Shiraz E-Medical Journal.

[ref10] Glanz K, Rimer B. K, Viswanath K (2008). Health behavior and health education: theory, research, and practice. John Wiley & Sons.

[ref11] Green L. W, Kreuter M. W (2005). Health program planning: An educational and ecological approach.

[ref12] Gysels M, Richardson A, Higginson I. J (2004). Communication training for health professionals who care for patients with cancer: a systematic review of effectiveness. Supportive Care in Cancer.

[ref13] Hauer K. E, Carney P. A, Chang A, Satterfield J (2012). Behavior change counseling curricula for medical trainees: a systematic review. Academic Medicine.

[ref14] Kurtz S, Silverman J, Benson J, Draper J (2003). Marrying content and process in clinical method teaching: enhancing the Calgary-Cambridge guides. Academic Medicine.

[ref15] Kurtz S. M (2002). Doctor-patient communication: principles and practices. The Canadian Journal of Neurological Sciences.

[ref16] Laidlaw T. S, Kaufman D. M, Macleod H, Sargeant J, Langille D. B (2001). Patients’satisfaction with their family physicians’ communication skills: a Nova Scotia survey. Academic Medicine.

[ref17] Lang F, Everett K, McGowen R, Bennard B (2000). Faculty Development in Communication Skills Instruction: Insights from a Longitudinal Program with “Real-time Feedback”. Academic Medicine.

[ref18] Loureiro E, Severo M, Bettencourt P, Ferreira M. A (2011). Third year medical students’ perceptions towards learning communication skills: Implications for medical education. Patient Education and Counseling.

[ref19] Merckaert I, Libert Y, Razavi D (2005). Communication skills training in cancer care: where are we and where are we going?. Current opinion in Oncology.

[ref20] Montini T, Noble A. A, Stelfox H. T (2008). Content analysis of patient complaints. International Journal for Quality in Health Care.

[ref21] Rees C, Sheard C, McPherson A (2004). Medical students’ views and experiences of methods of teaching and learning communication skills. Patient Education and Counseling.

[ref22] Sharma M, Romas J. A (2011). Theoretical foundations of health education and health promotion. Jones & Bartlett Publishers.

[ref23] Shumway J, Harden R (2003). AMEE Guide. No. 25: The assessment of learning outcomes for the competent and reflective physician. Medical Teacher.

[ref24] Teutsch C (2003). Patient–doctor communication. Medical Clinics of North America.

